# Parental vaccine hesitancy in Brazil: results from a household survey

**DOI:** 10.1590/0102-311XEN195724

**Published:** 2025-07-21

**Authors:** Ana Isabel do Nascimento, Danilo dos Santos Conrado, Lisany Krug Mareto, Micael Viana de Azevedo, João Cesar Pereira da Cunha, Gabriel Serrano Ramires Koch, Laysa Gomes Osório, Samara Tessari Pires, Letícia Suemi Arakaki, Sara Raquel Pinto Borges, Robson França Gomes e Silva, Rodrigo Mayer Pucci, João Guilherme de Novaes Corrêa, João Vitor Barrio, Maria Eduarda de Souza Rodrigues, Márcio José de Medeiros, Ana Paula Sayuri Sato, Maria Elizabeth Araújo Ajalla, Cláudia Du Bocage Santos Pinto, Everton Falcão de Oliveira

**Affiliations:** 1 Universidade Federal de Mato Grosso do Sul, Campo Grande, Brasil.; 2 Universidade Federal do Rio de Janeiro, Rio de Janeiro, Brasil.; 3 Faculdade de Saúde Pública, Universidade de São Paulo, São Paulo, Brasil.

**Keywords:** Vaccination Hesitancy, Immunization, COVID-19, Hesitação Vacinal, Imunização, COVID-19, Vacilación a la Vacunación, Inmunización, COVID-19

## Abstract

Vaccine-hesitant parents delay or refuse their children’s immunization and constitute a significant public health concern. Our study aims to measure parental vaccination hesitancy and its associated factors in parents residing in Campo Grande, Mato Grosso do Sul State, Brazil. From September 2022 to October 2023, a cross-sectional study aligned to a household survey was carried out to measure vaccination coverage in Campo Grande. The two-stage cluster sampling proposed by the World Health Organization to estimate vaccination coverage was adopted in this study. All residing parents of children aged under 12 years were included. Data were collected by face-to-face interviews using the SAGE Working Group questionnaire to assess parental vaccine hesitancy. We classified the reasons for hesitancy under the 3C conceptual model of vaccine hesitancy determinants. Descriptive statistics characterized the study population and a univariate and multivariate logistic regression assessed the association between hesitancy and other study variables. This study included 158 parents, 39.2% of whom hesitated to get their children immunized. COVID-19 vaccines produced the greatest hesitancy (77.4%). Participants mentioned lack of confidence as the most common motive for their hesitancy (85.5%). Hesitant parents resided in bigger households (aOR = 1.31; 95%CI: 1.02; 1.72), believed there were reasons for not immunizing children (aOR = 4.02; 95%CI: 1.41; 12.77), and hesitated to get their own vaccines (aOR = 3.74; 95%CI: 1.80; 8.16). Results suggest an association of parental hesitancy with socioeconomical and behavioral factors.

## Abstract (pt)

Os pais hesitantes em vacinar atrasam ou recusam a imunização de seus filhos e causam uma preocupação significativa à Saúde Pública. Nosso estudo busca mensurar a hesitação parental em vacinar e seus fatores associados entre pais residentes em Campo Grande, Mato Grosso do Sul, Brasil. Um estudo transversal alinhado a um inquérito domiciliar foi realizado entre setembro de 2022 e outubro de 2023 para mensurar a cobertura vacinal em Campo Grande. Este estudo adotou a amostragem por conglomerados em dois estágios proposta pela Organização Mundial da Saúde para estimar a cobertura vacinal, incluindo todos os pais de crianças menores de 12 anos no município. Nossos dados foram coletados por entrevistas pessoais usando o questionário do Grupo de Trabalho SAGE para avaliar a hesitação dos pais em vacinar. Classificamos as razões para a hesitação sob o modelo conceitual 3C de determinantes da hesitação vacinal. Uma estatística descritiva caracterizou a população do estudo e regressões logística univariada e multivariada avaliaram a associação entre hesitação e outras variáveis do estudo. Este estudo incluiu 158 pais, 39,2% dos quais hesitaram em imunizar seus filhos. As vacinas contra a COVID-19 foram as mais evitadas (77,4%) e falta de confiança, o motivo mais mencionado (85,5%). Os pais hesitantes residiam em famílias maiores (RCa = 1,31; IC95%: 1,02; 1,72), acreditavam haver motivos para não imunizar crianças (RCa = 4,02; IC95%: 1,41; 12,77) e hesitaram em tomar as próprias vacinas (RCa = 3,74; IC95%: 1,80; 8,16). Os resultados sugerem uma hesitação parental associada a fatores socioeconômicos e comportamentais.

## Abstract (es)

Los padres renuentes a la vacunación retrasan o se niegan a vacunar a los niños y son un importante problema de salud pública. Nuestro estudio tiene como objetivo medir la vacilación parental ante las vacunas y sus factores asociados entre los padres residentes de la ciudad de Campo Grande, estado de Mato Grosso do Sul, Brasil. De septiembre de 2022 a octubre de 2023 se realizó un estudio transversal alineado a una encuesta de hogares para medir la cobertura de vacunación en Campo Grande. En el estudio se adoptó el muestreo por conglomerados en dos etapas, propuesto por la Organización Mundial de la Salud para estimar la cobertura de vacunación. Se incluyeron en el estudio todos los padres residentes con niños menores de 12 años. Nuestros datos se recogieron a través de entrevistas presenciales utilizando el cuestionario del Grupo de Trabajo SAGE, para evaluar la vacilación parental ante las vacunas. Clasificamos las razones de vacilación según el modelo conceptual 3C de los determinantes de la reticencia a la vacuna. Para caracterizar la población de estudio se empleó estadística descriptiva y se realizó una regresión logística univariada y multivariada para evaluar la asociación entre la vacilación y otras variables del estudio. Un total de 158 padres fueron incluidos en el estudio, y el 39,2% dudó en vacunar a sus hijos. Las vacunas contra la COVID-19 provocaron más vacilaciones (77,4%), y la falta de confianza fue el motivo de vacilación más mencionado (85,5%). Los padres indecisos residían en hogares más grandes (ORa = 1,31; IC95%: 1,02; 1,72), creían que había razones para no inmunizar a los niños (ORa = 4,02; IC95%: 1,41; 12,77), y mostraron vacilaciones respecto a vacunarse (ORa = 3,74; IC95%: 1,80; 8,16). Los resultados sugieren que la vacilación de los padres está asociada con factores socioeconómicos y conductuales.

## Background

Preventing the deaths of newborns and children configures a key target of the 2030 Sustainable Development Goals under the broader objective of ensuring healthy lives at all ages ^1^. Advances in health over recent decades have reduced child mortality by more than 50% ^2^. Despite clean water, no other measure has effectively decreased mortality as vaccination ^2^ ^,^ ^3^. Therefore, childhood immunization is essential to build collective immunity, prevent children from becoming vulnerable to vaccine-preventable diseases, and achieve the 2030 Sustainable Development Goals ^4^.

In Brazil, over 15 vaccines are included in the basic vaccination schedule for children aged under 10 years ^5^. The Brazilian National Immunization Program (PNI, acronym in Portuguese) provide these vaccines free of charge and health units in the Brazilian Unified National Health System (SUS, acronym in Portuguese) offer them ^6^. Despite its past success in achieving high vaccine coverage rates, recent trends show a decline in vaccination rates across all Brazilian regions ^7^ ^,^ ^8^.

Reasons for vaccination delay and refusal can vary over time and locations. Amidst the factors that contributed to the decrease in vaccination, vaccine hesitancy became a major health issue and has been strongly associated with the decrease of vaccine uptake ^9^.

Vaccination hesitancy is defined as a “*motivational state of being conflicted about, or opposed to, getting vaccinated*” ^10^ (p. 213). This highly complex, volatile, and context-related phenomenon involves many determinants ^11^ ^,^ ^12^. Parents who alter vaccination schedules or refuse one or more vaccines are considered vaccine-hesitant parents ^13^. Their decisions regarding their children’s vaccinations configure a significant public health concern ^14^, entailing the understanding of the parental reasons for refusing to vaccinate their children to effectively address vaccination hesitancy ^13^ ^,^ ^15^. This study aims to assess vaccination hesitancy and find its determinants in parents residing in Campo Grande, a state capital in the Brazilian Center-West.

## Methods

### Study design and period

This cross-sectional study assessed childhood vaccine hesitancy in residents who participated in a population-based household survey in Campo Grande from September 2022 to October 2023. The household survey mainly aimed to estimate overall vaccine coverage (for all vaccines in the general population). Thus, the assessment of the prevalence of parental vaccination hesitancy was a secondary objective in the greater project.

### Sampling

A stratified two-stage cluster sampling design following the World Health Organization (WHO) guidelines was carried out ^16^. Sampling was conducted based on the number of households to be visited per cluster. Thus, determining the number of respondents, parents, and children, was avoided during the process. This sampling method aims to estimate vaccination coverage and is based on two stages: (1) cluster selection and (2) household selection.

(1) Definition and selection of clusters: assuming that the expected average vaccination coverage in Campo Grande for all vaccines available in the PNI totals 90% - with a confidence interval around 8% (i.e., 90% ± 8% coverage estimate) and an alpha (type I error) of 5% -, the effective sample size based on a simple random sampling totaled 101. A pilot study was carried out to determine the average number of people eligible for this study (individuals aged 12 years or above). After the pilot study, the average number of respondents per cluster in a three-hour interval with a field team of six researchers distributed in pairs totaled 10. Assuming a 0.33 intracluster correlation set the design effect size at three. The estimated number of clusters totaled 30 following the appropriate equation.

The clusters were chosen by simple random sampling using the cartographic base of census sectors from the Brazilian Institute of Geography and Statistics (IBGE, acronym in Portuguese) ^17^. The cluster in which the pilot study was carried out was included in this study. Therefore, an additional 29 clusters were then sampled. Clusters that primarily contained institutionalized populations (prisons and long-term care facilities such as nursing homes) were immediately replaced when drawn. Clusters containing large condominiums or gated communities that did not allow the entry of the study team to collect data after the initial contact by the researchers were also replaced.

(2) The definition of the number and selection of the households was based on the pilot study. The number of visited households to find an eligible participant averaged 1.5, the inflation factor to account for refusals and non-respondent residences totaled 1.05, and the average number of respondents per day of data collection equaled 10. Therefore, the number of households per cluster was defined as 15.

Those households were selected by random simple probabilistic sampling. The random sampling and spatial allocation of clusters and households were performed using the *sf* package on R, 3.4.2 (http://www.r-project.org).

### Study population and data collection

All residents in Campo Grande who consented to participate in the household survey and reported having children aged under 12 years were eligible for this study. Data were collected by an interview using the SAGE Working Group questionnaire ^18^, which has been translated into Portuguese and linguistically adapted to fit our context following its original meaning ^12^ ^,^ ^19^. The translation and adaptation were verified in our sociocultural context in the pilot study. These data included socioeconomic and demographic characteristics, access to health units, vaccination services for children and infants, and questions related to parental perception about vaccines and vaccine hesitance following the WHO questionnaire.

The collected socioeconomic and demographic data included parents’ age, education (in years of study), number of residents per household, sex, ethnicity (white, mixed-race, black, Indigenous, or Asian, according to the IBGE criteria), income, access to drinking water (yes or no), access to sanitary sewer treatment (yes or no), and number of children (1, 2, and 3 or more). Income was categorized as low and non-low based on criteria of the Brazilian Federal Government for receiving social benefits, which classifies low-income individuals as those with a monthly per capita income equal to or below half of the national minimum wage.

During the interviews, participants were introduced to the concept of parental vaccination hesitancy (as defined by the WHO, which served as the basis to develop the data collection instrument) ^18^. Participants were then asked about their attitudes toward vaccination, including any hesitancy they may have had regarding their decision on their children’s vaccinations, along with the respective reasons for their hesitancy. The reasons for vaccine hesitancy were categorized according to the 3C conceptual model of vaccine hesitancy, which classifies determinants under confidence (lack of confidence in the vaccination and related subjects, such as information or health services), convenience (lack of convenience to access vaccination services), and complacency (low perception of the risk of vaccine-preventable diseases and lack of given importance to vaccination) ^18^. When applicable, the reasons for hesitancy were also examined under the light of “risk calculation” (individual assessments of the risks and benefits of vaccination) and “collective responsibility” (desire to protect others with one’s immunization status) from the 5C model of vaccine hesitancy ^20^.

### Statistical analysis

Descriptive statistics were used to characterize the study population. The data were analyzed based on vaccine hesitancy, which divided the study population into hesitant and non-hesitant parents. Continuous variables were reported as mean values and standard deviation (SD) and were compared with the Welch’s t-test. Categorical variables were reported as frequencies and compared in the univariable stage using the chi-squared and/or Fisher’s exact tests when applicable.

Binomial logistic regression models were used to evaluate the association between vaccine hesitancy and the covariates assessed in our study. This process was conducted in two steps: (1) inclusion of variables with a p-value < 0.20 in the univariate analysis to assess their association with the outcome and (2) application of the stepwise algorithm (considering both backward and forward) using the Akaike information criterion (AIC) to select the variables from step 1, control for potential confounding factors, and define the best-fitting model. Multicollinearity was assessed using the variance inflation factor (VIF). The Hosmer-Lemeshow test was used as goodness-of-fit measures for the model.

A 5% significance level was adopted for all hypothesis (α = 0.05). Analyses were performed on R, version 4.3.2, using the following packages: *tidyverse*, *descr*, and *generalhoslem*.

## Results

The survey found 164 individuals to be parents or legal guardians for children aged under 12 years, 158 of whom answered our questionnaire concerning parental vaccination hesitancy.

Most participants were self-reported mixed-race (88/158; 55.7%) women (111/158; 70.3%) with a mean age of 36.4 years (SD = 11.3). Average households had 3.9 individuals (SD = 1.4). Most respondents reported having one child under their care (74/158; 46.8%) with low income (87/158; 55.1%) and access to drinking water (152/158; 96.2%) and sanitary sewer services (81/158; 51.3%). Additionally, a substantial proportion lacked access to health insurance (117/158; 74.1%). The average educational attainment of the study population totaled 10.9 years (SD = 3.5) (Table 1).

**Table 1:** Study data according to the occurrence of vaccine hesitancy. Campo Grande, Mato Grosso do Sul State, Brazil, 2024.

Variables	Total (N = 158)	Hesitant parents (n = 62)	Non-hesitant parents (n = 96)	OR (95%CI)	p-value
	Mean (SD)	Mean (SD)	Mean (SD)		
Age	36.4 (11.3)	36.0 (8.7)	36.6 (12.7)	1.00 (0.98; 1.03)	0.716
Years of study	10.9 (3.5)	10.9 (3.7)	10.8 (3.34)	1.00 (0.91; 1.09)	0.941
Residents per household	3.9 (1.4)	4.19 (1.3)	3.77 (1.36)	0.79 (0.62; 1.01)	0.051
	n (%)	n (%)	n (%)		
Sex					0.294
Female	111 (70.3)	47 (75.8)	64 (66.7)	Reference	
Male	47 (29.7)	15 (24.2)	32 (33.3)	1.56 (0.76; 3.28)	
Ethnicity					0.820
White	45 (28.5)	18 (29.0)	27 (28.1)	Reference	
Mixed-race	88 (55.7)	34 (54.8)	54 (56.2)	0.94 (0.45; 1.99)	
Black	13 (8.2)	4 (6.5)	9 (9.4)	0.68 (0.16; 2.50)	
Others *	12 (7.6)	6 (9.7)	6 (6.3)	1.49 (0.39; 5.63)	
Low income					0.906
Yes	87 (55.1)	35 (56.5)	52 (54.2)	Reference	
No	71 (44.9)	27 (43.5)	44 (45.8)	1.10 (0.57; 2.10)	
Access to drinking water					0.680
Yes	152 (96.2)	59 (95.2)	93 (96.9)	Reference	
No	6 (3.8)	3 (4.8)	3 (3.12)	0.64 (0.11; 3.81)	
Access to sanitary sewer treatment					0.816
Yes	81 (51.3)	33 (53.2)	48 (50.0)	Reference	
No	77 (48.7)	29 (46.8)	48 (50.0)	1.14 (0.60; 2.17)	
Access to health insurance					0.555
Yes	41 (25.9)	14 (22.6)	27 (28.1)	Reference	
No	117 (74.1)	48 (77.4)	69 (71.9)	0.75 (0.35; 1.57)	
Children					0.591
1	74 (46.8)	26 (41.9)	48 (50.0)	Reference	
2	50 (31.6)	22 (35.5)	28 (29.2)	0.69 (0.33; 1.45)	
3 or more	34 (21.5)	14 (22.6)	20 (20.8)	0.77 (0.33; 1.81)	
Do you believe that vaccines can protect yourself and children from serious diseases?					0.152
Yes	156 (98.7)	60 (96.8)	96 (100.0)	Reference	
No	2 (1.2)	2 (3.2)	0 (0.0)	‒	
Do you believe that there are reasons for people to not get vaccinated?					0.004
Yes	20 (12.7)	14 (22.6)	6 (6.32)	Reference	
No	137 (87.3)	48 (77.4)	89 (93.7)	4.23 (1.57; 12.8)	
Have any of the following factors - distance, opening hours of the health care facility, time required to get to the health facility, waiting time at the health unit - prevented you from getting vaccinated?					0.603
Yes	46 (29.1)	20 (32.3)	26 (27.1)	Reference	
No	112 (70.9)	42 (67.7)	70 (72.9)	1.28 (0.63; 2.58)	
Have you ever received or heard negative information about vaccination?					0.439
Yes	87 (55.1)	37 (59.7)	50 (52.1)	Reference	
No	71 (44.9)	25 (40.3)	46 (47.9)	1.36 (0.71; 2.62)	
Which source of information do you primarily use to learn about vaccination-related information?					0.301
Health care workers/websites or profiles from official healthcare organizations	38 (24.1)	10 (16.1)	28 (29.2)	Reference	
Traditional news (official website or television)	61 (38.6)	27 (43.5)	34 (35.4)	0.46 (0.18; 1.09)	
Social media	46 (29.1)	20 (32.3)	26 (27.1)	0.47 (0.18; 1.18)	
Others (friends, neighbors, or none)	13 (8.2)	5 (8.1)	8 (8.3)	0.58 (0.15; 2.36)	
Have you ever been advised by healthcare providers about vaccination?					0.537
Yes	145 (91.8)	59 (95.2)	86 (89.6)	Reference	
No	8 (5.1)	2 (3.2)	6 (6.2)	1.96 (0.42; 15.2)	
Do not know/do not remember	5 (3.1)	1 (1.6)	4 (4.2)	2.48 (0.33; 69.0)	
How would you describe your relationship with the healthcare workers at the health unit you attend?					0.177
Great/Good	105 (66.5)	39 (62.9)	66 (68.8)	Reference	
Reasonable/Indifferent	31 (19.6)	17 (27.4)	14 (14.6)	0.49 (0.21; 1.11)	
Bad	10 (6.3)	2 (3.2)	8 (8.3)	2.23 (0.51; 16.9)	
Do not attend any health care unit	12 (7.6)	4 (6.5)	8 (8.3)	1.16 (0.33; 4.75)	
Did you ever hesitate to get yourself vaccinated?					< 0.001
Yes	95 (60.1)	49 (79.0)	46 (47.9)	Reference	
No	63 (39.9)	13 (21.0)	50 (52.1)	4.03 (1.98; 8.68)	

95%CI: 95% confidence interval; OR: odds ratio.

* Self-declared Indigenous and Asian were grouped due to their low occurrence.

Overall, 39.2% of parents (62/158) reported having hesitated to get their children vaccinated. In comparison, a larger proportion of parents reported hesitancy to vaccinate themselves (95/158; 60.1%). Participants reported most often that COVID-19 vaccines caused hesitancy (48/62; 77.4%), followed by the influenza vaccine (5/62; 8.1%). Additionally, 12.9% (8/62) of participants were unable to recall which specific vaccines their children had missed. Less frequently mentioned vaccines included the diphtheria, tetanus, and pertussis; oral poliovirus; inactivated poliovirus; yellow fever, herpes zoster, and HPV vaccines, each being mentioned by only one parent (1/62; 1.61%).

Table 2 shows the reasons for vaccine hesitancy. The primary reasons for vaccine hesitancy were predominantly related to lack of confidence (53/62; 85.5%). The most frequently cited concerns included the perception that vaccines were unsafe and worries about potential side effects (39/62; 62.9%), exposure to negative media coverage about vaccines (33/62; 53.2%), doubts about the effectiveness of vaccines (19/62; 30.6%), and negative opinions about vaccine safety from others (15/62; 24.2%). Other reasons included negative experiences with previous vaccines (15/62; 24.2%), reports of adverse experiences with vaccines from acquaintances (7/62; 11.3%), and fear of needles (2/62; 3.2%). Over 80% of these reasons referred to the COVID-19 pandemic - apart from fear of needles, which was reported by 50% (1/2) of those who mentioned it.

**Table 2:** Parental reasons for vaccination hesitancy. Campo Grande, Mato Grosso do Sul State, Brazil, 2024.

3C	Parental reported reason	n (%)
Confidence	Did not think the vaccine was safe/concerned about side effects	39 (62.9)
	Have heard or read negative media about vaccines	33 (53.2)
	Did not think the vaccine was effective *	19 (30.6)
	Someone else told me that the vaccine was not safe	15 (24.2)
	Had a bad experience or reaction to a previous vaccination	15 (24.2)
	Someone else told me they/their child had a bad reaction	7 (11.3)
	Fear of needles *	2 (3.2)
Complacency	Did not think it was needed **	18 (29.0)
	Forgetfulness	3 (4.8)
	Religious reasons **	2 (3.2)
Convenience	Did not know where to get good/reliable information	12 (19.4)
	Not possible to leave other work (at home or other)	6 (9.7)
	Lack of time	3 (4.8)
	Did not know where to get vaccination	1 (1.6)
	Travel	1 (1.6)

Note: according to the 5C conceptual model of vaccination hesitancy determinants, the items marked with * can also be comprehended under the “risk calculation” dimension, whereas those marked with ** can be comprehended under the “collective responsibility” dimension.

Complacency reasons for vaccine hesitancy included forgetfulness (3/62; 4.8%) and the perception that vaccines were unnecessary (18/62; 29%). Notably, participants predominantly reported such belief regarding the COVID-19 pandemic (15/18; 83%). Lastly, convenience-related reasons for vaccine hesitancy included not knowing where to find good or reliable information about vaccines (12/62; 19.4%), being unable to take time off work to vaccinate their children (6/62; 9.7%), lack of time (3/62; 4.8%), not knowing where to obtain the vaccine, and being away from home during the vaccination schedule (1/62; 1.6% each). Among these convenience-related reasons, participants reported not knowing where to find good information about vaccines (11/12; 91.7%), lack of time (1/6; 33.3%), and being unable to take time off work (2/6; 33.3%) regarding the COVID-19 pandemic.

Regarding access to vaccination and vaccination services, 29.1% (46/158) of respondents reported difficulties accessing vaccination for their children. Among those, 60.9% (28/46) described obstacles related to the lack of vaccines. Additionally, 26.1% (12/46) reported difficulties related to the operating hours of health facilities, 21.7% mentioned issues with waiting times at these facilities, and 19.6% found difficulties related to the distance to healthcare facilities. Moreover, 8.9% (14/158) of parents reported other reasons for delaying or refusing vaccines. Of these, 28.6% (4/14) reported other access difficulties, 42.9% (6/14) mentioned health conditions and medical contraindications, whereas other reasons included opposition to vaccination and lack of support from spouses in taking their children to healthcare facilities.

Regarding the relationship with healthcare workers at the attended health facilities, 19.6% (31/158) of parents reported a reasonable or indifferent relationship and 6.3% (10/158), a poor relationship. Jointly, 66.5% (105/158) reported a great or good relationship with healthcare workers. Almost all participants reported receiving advice during pregnancy or prenatal care on the importance of vaccinating their children (145/158; 91.8%). A significant proportion of parents believed that most parents of children like theirs did not complete the full schedule of vaccines recommended by the PNI (98/158; 62%). Additionally, most participants did not change their beliefs about vaccination following the onset of the COVID-19 pandemic (130/158; 82.3%).

Most participants perceived no reasons for parents avoiding to vaccinate their children (137/158; 87.3%). However, hesitant parents were more likely to believe in reasons for avoiding vaccinating their children (p = 0.006). Primary reasons included a lack of public trust in the vaccines (11/20; 55%) (predominantly reported by hesitant parents), whereas non-hesitant parents mentioned illnesses and medical contraindications (6/20; 30%) more frequently. Notably, participants reported many of these reasons after the beginning of the COVID-19- pandemic (11/20; 55%).

Regarding other community-level vaccination aspects, more than half of the parents did not believe that parents from ethnic and religious groups faced difficulties vaccinating their children (90/158; 57%). Of those who perceived such difficulties (46/158; 29.1%), the predominant reason referred to the parental choice to forgo vaccination (35/46; 76.1%). Other less frequently mentioned reasons included insufficient outreach by healthcare facilities (9/46; 19.6%) and a lack of welcoming at healthcare facilities (2/46; 4.3%). Additionally, most parents found no discouragement from community leaders regarding vaccines for infants and children (130/158; 82.3%). Of the 10.1% (16/158) who experienced such discouragement, 37.5% (6/16) suffered the influence of religious leaders, 31.3% (5/16) political leaders, and 25.0% (4/16) healthcare workers.

Nevertheless, the great majority of participants believed vaccines could protect children from illnesses (156/158; 98.7%). Additionally, the hesitant parents changed their opinion significantly more due to the COVID-19 pandemic than non-hesitant parents (p = 0.021).

The main source of information for parents regarding infant and child vaccination referred to traditional media, including online platforms and television (61/158; 38.6%), followed by social media (46/158; 29.1%), and professional healthcare providers or health organization websites (38/158; 24.1%). More than half of parents reported having heard or read negative information about child vaccination (87/158; 55.1%), receiving most of which after the onset of the COVID-19 pandemic (58/87; 66.7%). Additionally, hesitant parents suffer significantly more influence from such information than non-hesitant parents (p < 0.001).

Variables with p-value smaller than 0.20 refer to number of residents per household, believing that vaccines can protect themselves and children from serious illnesses, believing in reasons for people getting no immunization, relationship with healthcare providers, and self-reported hesitancy. However, the covariates remaining in the final logistic regression model (Table 3) include number of residents per household, positive belief in reasons for people getting no vaccination, and positive parental self-reported hesitancy. All variables included in the final model were positively associated with parental vaccine hesitancy. The p-value for the Hosmer-Lemeshow test totaled 0.597, indicating a good model fit, whereas the low VIF suggested no multicollinearity in the model.

**Table 3:** The final model for the occurrence of vaccine hesitancy (outcome = vaccine hesitancy). Campo Grande, Mato Grosso do Sul State, Brazil, 2024.

Covariates	β coefficient (SE)	VIF	Adjusted OR (95%CI)
Intercept	-2.54 (0.64)	-	0.08 (0.02; 0.26)
Residents per household	0.27 * (0.13)	1.01	1.31 (1.02; 1.72)
Are there any reasons you think children should not be vaccinated? (Yes)	1.39 * (0.55)	1.01	4.02 (1.41; 12.77)
Did you hesitate to get yourself vaccinated? (Yes)	1.32 ** (0.38)	1.00	3.74 (1.80; 8.16)

95%CI: 95% confidence interval; OR: odds ratio; SE: standard error; VIF: variance inflation factor.

* p-value < 0.050;

** p-value < 0.001.

## Discussion

This study found a higher estimated prevalence of parental vaccination hesitancy than that in other studies carried out in Brazil and other countries prior to the pandemic ^21^ ^,^ ^22^ ^,^ ^23^ ^,^ ^24^. Specifically, participants mentioned COVID-19 vaccines most often as a source of hesitancy, as 30.4% of parents expressed reluctance to immunizing their children. This rate of hesitancy exceeds that observed in other COVID-19 vaccine-specific studies in Brazil ^25^ ^,^ ^26^.

Brazil experienced significant political turbulence during the pandemic, which may have adversely affected its COVID-19 vaccination rates ^27^ ^,^ ^28^. Our findings suggest that the higher prevalence of COVID-19-specific vaccine hesitancy is more likely attributable to behavioral resistance rather than socioeconomic factors, a pattern also observed by Gramacho et al. ^27^. Consistent with the literature, the reasons for vaccine hesitancy in our study primarily focused on concerns about immunization safety, potential side effects, low perceived risk of illnesses, questions about vaccination efficacy, and other safety-related concerns ^13^.

The television emerged as the main source of information for parents, which may increase the positive exposure reinforcement about vaccination news and campaigns in traditional media ^27^ ^,^ ^29^. A significant percentage of hesitant parents suffered the influence of negative media coverage regarding vaccination, particularly following the onset of the pandemic. The COVID-19 infodemic may have adversely affected vaccination efforts in Brazil as the widespread dissemination of misinformation in the period likely undermined confidence in COVID-19 immunization ^30^. However, receiving positive recommendations and reliable information from healthcare providers, friends, and family may enhance the acceptance of vaccination ^31^ ^,^ ^32^.

The increase in the number of residents per household was significantly associated with a rise in vaccine hesitancy. This pattern has been observed in other studies: participants residing in larger households, particularly those with six or more residents, were more likely to express unwillingness or hesitancy to receive COVID-19 vaccination ^33^. Similarly, parents in households with more than four children hesitated more toward vaccinating their infants ^23^. This finding is particularly concerning as bigger households often face greater challenges in adhering to preventive measures, such as isolation and social distancing. Additionally, larger households are frequently associated with lower income levels, which may offer logistical challenges in accessing vaccination services.

Another aspect related to social determinants of health that may have influenced the high vaccine hesitancy in the parents in this study refers to low income prevalence, as most participants reported earning less than half a minimum wage. Living conditions and income levels significantly influence access to resources and impact overall quality of life and health outcomes. The COVID-19 pandemic increased inequalities and may have adversely affected vaccine uptake ^34^.

Our study found a positive association between adult vaccine hesitancy and parental hesitancy in immunizing their children. A web-based survey in China suggested that adult vaccine hesitancy could be directly or indirectly associated with childhood immunization as hesitant adults were less inclined to vaccinate their children against COVID-19 ^35^. However, adult hesitancy exceeded parental hesitancy possibly because adult vaccines were approved before childhood vaccines in the country and many adults had to get vaccinated against COVID-19 to continue working during the pandemic.

We observed a low prevalence of hesitancy toward routine immunizers the PNI recommends as a high percentage of parents believed vaccines could prevent illnesses and protect their children. This may be attributed to the significant success of the PNI in controlling many vaccine-preventable diseases, such as rubella, polio, and neonatal tetanus. Over the years, this program has substantially reduced infant mortality and hospitalization rates in the country, developing a strong culture of immunization ^36^ ^,^ ^37^ ^,^ ^38^.

Nevertheless, a significant percentage of participants believed that other parents avoided vaccinating their children, a perception they held even before the pandemic. Moreover, most participants perceived the non-vaccination of certain religious and ethnic groups as a self-chosen decision. Vaccination, understood as a social contract, is reinforced when immunization is perceived as a moral obligation and can serve as an effective communication tool to increase vaccine uptake ^39^. However, individual adherence to this social contract may depend on the wider community’s acceptance of vaccination. If non-vaccination becomes common and socially accepted, it could impede vaccine uptake ^39^ ^,^ ^40^.

We emphasize that vaccine hesitancy constitutes an influential factor that hinders vaccine uptake and impacts vaccination coverage ^41^. Therefore, enhancing vaccination acceptance requires acknowledging hesitancy in the community toward a multidisciplinary approach. Building a healthy and empathic partnership and relationship between healthcare providers and the community builds confidence in finding hesitancy among patients. Interventions via collective and individual honest and focused discussions may offer spaces to properly advise hesitant parents with evidence-based information and evoke confidence to promote real-life changes ^42^. Likewise, health communication for the community may target the engagement of the population and positive messages focused on the benefits of the vaccination, previously testing such communication to the meet community worries ^43^. Nevertheless, an individual approach and a real and empathic understanding of the needs and apprehensions of the parents constitute the foundation to strengthen motivation toward acceptance ^42^ ^,^ ^43^.

Finally, our study has certain limitations, such as its relatively small sample of parents. We also faced difficulties accessing a higher income and educational layer of the municipality as two of the higher income clusters had to be replaced given the lack of access of the researchers to the area (closed gates communities), thus possibly making our sample a more homogeneous one. Still, we carried out a population-based survey in a Brazilian state capital and could assess parental vaccine hesitancy during a transitional period of the pandemic that eased social isolation measures, enabling face-to-face interviews with participants.

## Conclusions

Our study assessed the prevalence of vaccine hesitancy and its associated determinants. We observed a higher level of parental hesitancy than in other nationwide studies that was mainly related to COVID-19 vaccines, which participants primarily attributed to their lack of trust in these immunizations. Parental hesitancy affected routine vaccination less. Hesitancy was associated with socioeconomic factors such as the number of residents in the household and behavioral factors, including prior self-vaccine hesitancy and the belief that children should receive no immunization. Lastly, further studies are needed to better understand the impact of the pandemic on vaccination practices explore potential shifts in vaccine perceptions in parents, and describe the reasons behind the decline in routine vaccination coverage in Brazil.
